# Medial Meniscus Physiologic Extrusion Across Sitting, Bipedal, and Unipedal Stance: The Roles of Generalized Hypermobility and Patellar Tendon Stiffness

**DOI:** 10.3390/diagnostics16071000

**Published:** 2026-03-26

**Authors:** Koray Kaya Kilic, Nevfel Kahvecioglu, Mustafa Yalcin, Serkan Gurcan, Ozkan Kose

**Affiliations:** 1 Department of Radiology, University of Health Sciences, Antalya Education and Research Hospital, 07100 Antalya, Türkiye; koraykayakilic@yahoo.com (K.K.K.);; 2 Private Practice, Gurcan Orthopedics Clinic, Cevizlik, Bakırköy, 34365 Istanbul, Türkiye; 3 Department of Orthopedics and Traumatology, University of Health Sciences, Antalya Education and Research Hospital, 07100 Antalya, Türkiye

**Keywords:** medial meniscus extrusion, ultrasonography, weight bearing, generalized joint hypermobility, Beighton score, shear-wave elastography

## Abstract

**Background/Objectives**: Medial meniscus extrusion (MME) is a quantitative marker of altered meniscal containment and load sharing. Although ultrasonography enables dynamic assessment under functional loading, it remains unclear whether generalized ligamentous hypermobility influences physiologic extrusion behavior in healthy knees. The aim of this study was to quantify load-dependent MME in healthy adults and to determine whether generalized hypermobility is associated with greater physiologic extrusion under progressive loading conditions. **Methods:** In this prospective observational study, 106 healthy adults aged 18–40 years were evaluated between October and December 2025. Generalized joint hypermobility was defined as a Beighton score ≥5. MME was measured by standardized ultrasonography on the dominant limb in three conditions: sitting (unloaded), bipedal stance, and unipedal stance. Patellar tendon shear-wave elastography (SWE) was recorded in kilopascals (kPa). Interobserver reliability was assessed in the first 25 participants using ICC (2,1). Group comparisons, multivariable linear regression for loading-related Δ-extrusion (Unipedal−Sitting and Bipedal−Sitting), and a linear mixed-effects model for repeated MME measures, including a Position × Hypermobility interaction, were performed. **Results:** Twenty-eight participants (26.4%) were classified as hypermobile. The hypermobile group showed significantly lower patellar tendon SWE than controls (23.8 ± 7.0 vs. 37.6 ± 9.7 kPa, *p* < 0.001). MME increased stepwise with loading in both groups and remained consistently higher in hypermobile participants across sitting, bipedal, and unipedal conditions (all *p* < 0.001). Loading-related extrusion was also greater in the hypermobile group for both Bipedal−Sitting (*p* = 0.037) and Unipedal−Sitting (*p* = 0.002). In multivariable regression, lower patellar tendon SWE independently predicted greater loading-related extrusion, whereas hypermobility status did not remain an independent predictor. In the mixed model, the Position × Hypermobility interaction was significant and was most pronounced during the unipedal stance. **Conclusions:** In healthy adults, medial meniscus extrusion increases stepwise from unloaded sitting to bipedal and unipedal weight bearing. Participants with generalized hypermobility demonstrated higher physiologic MME values and a more pronounced load-dependent pattern, particularly during unipedal stance. However, in adjusted analyses, lower patellar tendon stiffness on SWE, rather than hypermobility status itself, independently predicted greater loading-related extrusion. These findings support a contextual interpretation of ultrasound-based MME measurements in relation to loading condition and hypermobility phenotype.

## 1. Introduction

The medial meniscus is a key secondary stabilizer of the knee and a major contributor to tibiofemoral load sharing, shock absorption, and joint congruity [1]. Under physiological conditions, the meniscus maintains hoop tension and distributes compressive forces over a broad contact area [2]. Disruption of this mechanism, whether due to degeneration, root tears, or insufficiency of meniscotibial attachments, may allow the meniscus to displace beyond the tibial margin. Medial meniscus extrusion (MME) refers to the radial displacement of the medial meniscal body past the edge of the tibial plateau. It is increasingly recognized as a biomechanical marker of compromised meniscal function. Even when structural meniscal damage is subtle, extrusion may reflect loss of meniscal containment, reduced contact area, increased cartilage stress, and a higher risk of progressive medial compartment osteoarthritis. Accordingly, MME has attracted attention not only as an imaging finding but also as a potential quantitative parameter that links meniscal integrity to joint mechanics [3,4,5,6,7].

MME can be assessed using magnetic resonance imaging (MRI) or ultrasonography (US) [8,9]. MRI is considered the reference modality because it provides a comprehensive evaluation of meniscal morphology, associated cartilage lesions, bone marrow abnormalities, and ligamentous injuries [3,5]. However, conventional MRI is typically performed in a static, unloaded supine position; therefore, it may underestimate meniscal displacement that becomes more apparent during weight bearing. In contrast, the US offers several practical advantages for extrusion assessment: it is widely available, inexpensive, free of ionizing radiation, allows rapid side-to-side comparison, and, most importantly, can be performed dynamically under different loading conditions [3,8,9,10]. These strengths make the US particularly suitable for investigating physiologic meniscal behavior during functional postures [11,12,13,14]. Nevertheless, despite the growing use of the US for MME measurement, the reported diagnostic cut-off values vary substantially across studies [15,16,17,18,19,20]. This variability likely reflects differences in patient characteristics, knee positioning, probe orientation/pressure, anatomical reference points, and, critically, whether measurements are obtained under loaded or unloaded conditions. Importantly, not all extrusions should be interpreted as structurally pathologic. In asymptomatic knees, a certain degree of meniscal displacement may represent a physiologic, load-responsive adaptation rather than failure of meniscal restraint, particularly when assessed dynamically under weight-bearing conditions.

In healthy individuals, the meniscal position is not fixed; it varies with joint loading and with subject-specific factors [2]. Prior work suggests that MME may differ by age, loading conditions, sex, and body mass index (BMI), underscoring that “normal” values are not uniform across populations [11,12,13,14,15,16,17,18,19,20]. At the same time, most studies have focused on structural or degenerative extrusion, whereas the physiologic range and load-responsive behavior of MME in asymptomatic knees remain insufficiently defined. This poses an important limitation for ultrasonographic interpretation, as dynamic MME measurements obtained under different loading conditions may be difficult to classify as physiologic or pathologic without a well-characterized reference pattern. One potentially relevant but insufficiently studied source of this physiologic variability is generalized ligamentous hypermobility. Hypermobility is associated with increased soft-tissue laxity and altered joint kinematics, and may therefore modify the functional restraint provided by capsulomeniscal and meniscotibial structures. Accordingly, the key unanswered question is not simply whether hypermobility is a distinct characteristic, but whether it meaningfully influences physiologic MME behavior under load and, in turn, affects the interpretation of ultrasound-based measurements in otherwise healthy knees.

The purpose of this study was to quantify medial meniscus extrusion in healthy adults across unloaded (sitting) and loaded (bipedal and unipedal stance) positions using standardized ultrasonography, and to determine whether generalized ligamentous hypermobility contributes to physiologic variability in load-dependent MME. We hypothesized that healthy adults with ligamentous hypermobility would exhibit greater physiologic extrusion of the medial meniscus than non-hypermobile controls, particularly under loaded conditions. By addressing this question, the study aimed to define a more coherent physiologic framework for interpreting dynamic US-based MME measurements in healthy knees, rather than treating hypermobility and extrusion as separate parallel issues.

## 2. Materials and Methods

### 2.1. Study Design and Participants

This prospective observational study was conducted between October 2025 and December 2025 at Antalya Training and Research Hospital and included a convenience sample of healthy adult volunteers aged 18 to 40 years. Eligibility was established based on the absence of clinical symptoms, the absence of suspicion for knee pathology on clinical assessment, and standardized orthopedic examination findings; however, no imaging-based exclusion was performed. Before enrollment, all participants underwent a standardized clinical assessment by an orthopedic surgeon. Demographic and anthropometric variables (age, sex, height, and weight) were recorded, and body mass index (BMI) was calculated. The dominant lower limb, defined as the leg naturally preferred for kicking a ball, was determined for each participant and used for all imaging measurements in order to standardize the assessment to a single functionally preferred side and minimize side-to-side methodological variability [21]. The physical examination included evaluation of lower-extremity alignment, including interepicondylar and intermalleolar distances, and assessment of knee and patellofemoral range of motion. A complete neurovascular examination and manual muscle strength testing were performed. Ligament stability was assessed using standard clinical tests of the medial and lateral collateral ligaments and the anterior and posterior cruciate ligaments. Meniscal examination included assessment of medial and lateral joint-line tenderness and McMurray testing; participants were required to have no clinical suspicion of meniscal pathology. In addition, generalized joint hypermobility was assessed using the Beighton score and recorded for all participants. All physical examination findings were required to be within normal limits. Participants were excluded if any abnormal clinical finding raised suspicion for an underlying knee pathology. Additional exclusion criteria were: history of inflammatory/rheumatologic disease, prior knee surgery for any reason, known connective tissue disorder (e.g., Ehlers–Danlos syndrome) other than hypermobility defined by Beighton score, and refusal to participate. Notably, participants with ligamentous laxity/hypermobility, as assessed by the Beighton score, were not excluded, provided all other eligibility criteria were met, since hypermobility was not considered a connective tissue disorder but rather an anatomic variation. The study was conducted in accordance with the Declaration of Helsinki, and written informed consent was obtained from all participants. The Institutional Review Board approved the study protocol (Approval Date: 6 November 2025, No: 19–20).

### 2.2. Sample Size Calculation

An a priori sample size calculation was performed with Δ-extrusion as the primary endpoint, defined as the change in medial meniscus extrusion from unloaded sitting to loaded single-leg stance (Unipedal − Sitting). Based on Achtnich et al. [13], the loading-related increase in medial meniscus extrusion (Δ-extrusion) in asymptomatic adults was 0.8 ± 0.6 mm; therefore, an SD of 0.6 mm was used for planning. Because no established minimal clinically significant difference exists for Δ-extrusion in healthy populations, a between-group difference of 0.4 mm was prespecified as clinically relevant, corresponding to approximately 50% of the physiological loading-related Δ-extrusion (0.8 mm) reported by Achtnich et al. [13]. Generalized joint hypermobility was defined as a Beighton score ≥5, using an established adult cutoff for the age range studied [22]. The Beighton score, a 9-point scale used to assess generalized joint hypermobility, uses age-specific cutoffs to account for age-related differences in joint laxity. Generalized joint hypermobility is typically defined as a Beighton score of ≥6/9 in pre-pubertal children and adolescents, ≥5/9 in adults up to 50 years of age, and ≥4/9 in adults older than 50 years. Given the expected low-to-moderate prevalence of Beighton ≥5 in asymptomatic adults and the possibility of enrolling fewer hypermobile individuals than anticipated, we planned for unequal group sizes [23]. We inflated the overall recruitment target to ensure an adequate number of hypermobile participants for the primary comparison and adjusted analyses. With a two-sided α = 0.05 and 80% power, the required sample size to detect a 0.4-mm difference (SD 0.6 mm) under an approximate 3:1 allocation was 24 hypermobile and 72 non-hypermobile participants (total *n* = 96). Allowing for 10% exclusions or incomplete/invalid ultrasound measurements, the planned recruitment target was 106 participants. The primary analysis was planned using a multivariable linear regression model to estimate the independent association between hypermobility and Δ-extrusion, adjusting for sex and BMI.

### 2.3. US Assessment of Medial Meniscus Extrusion and Patellar Tendon Elastography

**Figure 1:**
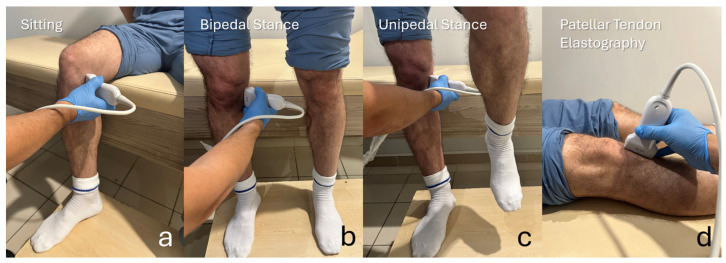
Ultrasound acquisition positions during meniscus extrusion measurements and patellar tendon elastography. Representative photographs illustrating the standardized examination setup. (**a**) Sitting (unloaded) position during ultrasound assessment (90° knee flexion, 90° hip flexion). (**b**) Bipedal stance (weight-bearing) position. (**c**) Unipedal stance (single-leg weight-bearing) position. (**d**) Patellar tendon elastography acquisition was performed with the transducer placed over the patellar tendon with the knee in full extension. All measurements were obtained on the dominant limb, with the probe orientation and anatomical landmarks held constant at each position.

Participants were assessed in three standardized conditions: sitting (unloaded), standing with bipedal weight bearing, and standing with unipedal weight bearing on the dominant limb (Figure 1). Measurements were obtained in a fixed, standardized order—sitting, followed by bipedal stance, then unipedal stance—rather than in a randomized order, to ensure consistency across participants and to reflect a progressive loading sequence. No formal rest or acclimatization period was used between positions; instead, measurements were performed sequentially once a stable posture was achieved. The average time required for assessment in each position was approximately 2 min.

**Figure 2:**
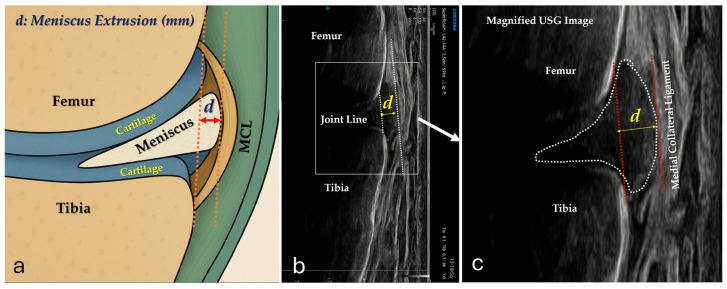
Definition and ultrasound measurement of medial meniscus extrusion (MME). (**a**) Schematic illustration of the medial compartment showing the femur, tibia, articular cartilage, medial meniscus, and medial collateral ligament (MCL). MME (d, mm) was defined as the horizontal distance between the peripheral border of the medial meniscus and a vertical reference line aligned with the outer cortical margin of the medial tibial plateau at the joint line. (**b**) Representative longitudinal ultrasound image of the medial joint line demonstrating the standardized probe position to visualize the femoral condyle, tibial plateau, and the joint line (reference line). (**c**) Example ultrasound image depicting the delineation of the medial meniscus (dotted contour) and the tibial reference line (red dashed line); the measured distance corresponds to MME (d, mm).

All ultrasound examinations were performed using an ultrasound system (Samsung V8 General Imaging, Samsung Healthcare, Seoul, Republic of Korea) equipped with a linear transducer (Samsung LA2-14A, 2–14 MHz, Samsung Healthcare, Seoul, Republic of Korea). Measurements were obtained by a radiologist with 10 years of experience in musculoskeletal ultrasound. Only the dominant limb was evaluated in each participant. Ultrasound images were obtained at the mid-medial aspect of the knee with the probe aligned parallel to the medial collateral ligament in a longitudinal orientation, thereby generating coronal-plane images [8,11,24]. The transducer was positioned on the mid-medial aspect of the knee, oriented perpendicular to the skin surface to minimize anisotropy and measurement error. After acquiring an optimal image, the meniscal margin and the relevant bony reference lines were identified, and extrusion was quantified using the system’s electronic caliper/distance measurement tool (Figure 2). To ensure that meniscal extrusion was assessed on the same longitudinal (coronal) section across subsequent standing conditions, a skin marker was placed at the joint line to standardize transducer placement.

**Figure 3:**
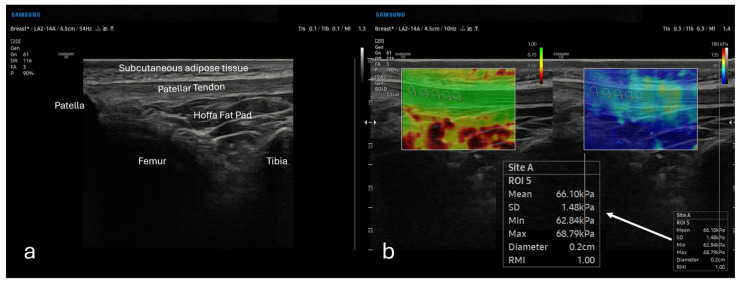
Ultrasound and shear-wave elastography assessment of the patellar tendon. (**a**) Longitudinal grayscale ultrasonographic image obtained at the anterior knee, demonstrating the patellar tendon and adjacent anatomical landmarks, including the patella, subcutaneous adipose tissue, Hoffa fat pad, femur, and tibia. (**b**) Shear-wave elastography (SWE) image of the patellar tendon with the elastography map overlaid on the B-mode image. Five 0.2 cm circular regions of interest (ROIs) placed along the tendon are shown for quantitative stiffness measurement (kPa). The arrow indicates the magnified quantitative SWE output (example ROI measurement).

Patellar tendon elastography was performed in the supine position (knee in full extension) using the same ultrasound platform and linear transducer (Figure 1). After identifying the patellar tendon on B-mode ultrasonography, images were obtained in a standardized longitudinal plane with the transducer aligned parallel to the tendon fibers to generate a consistent longitudinal (sagittal) section. A gel standoff pad (approximately 5 mm coupling layer) was used, and acquisitions were obtained with minimal probe compression to avoid pressure-related alterations in SWE values [25]. The SWE sampling box was positioned over the mid-portion of the patellar tendon, and stiffness was recorded in kilopascals (kPa). Five small, circular regions of interest (ROIs), each 0.2 cm in diameter, were placed within the tendon substance, carefully avoiding the paratenon and the adjacent Hoffa fat pad; the mean value across the five ROIs was used for analysis [26]. Measurements were accepted only when the elastogram and the quality/propagation map showed a stable, homogeneous pattern (Figure 3).

To standardize measurement reliability, the first 25 participants were independently evaluated by a second radiologist with 15 years of experience, and interobserver agreement was calculated. After confirming acceptable reliability (ICC > 0.800 for all measurements) (Table 1), subsequent measurements were performed solely by the primary radiologist.

### 2.4. Statistical Analysis

Continuous variables were summarized as mean ± standard deviation (SD) with range when normally distributed and as median (interquartile range, IQR) with range when non-normally distributed, and categorical variables as counts and percentages. Normality was assessed visually (histograms and Q–Q plots) and using the Shapiro–Wilk test. Between-group comparisons (hypermobility vs. non-hypermobility) were conducted using the independent-samples Student’s *t* test for normally distributed continuous variables and the Mann–Whitney U test for non-normally distributed variables; categorical variables were compared using the chi-square test (or Fisher’s exact test when appropriate). Primary loading-related outcomes were defined as Δ-extrusion values: Δ(Bipedal − Sitting) and Δ(Unipedal − Sitting). To examine independent associations with these outcomes, separate multivariable linear regression models were fitted for Δ-unipedal and Δ-bipedal extrusion, with age, BMI, sex, patellar tendon elastography (kPa), and hypermobility status (Beighton ≥ 5) entered simultaneously (enter method). Regression coefficients (B) with standard errors (SE), 95% confidence intervals (CI), and *p* values were reported. Model assumptions were checked using residual diagnostics, and multicollinearity was assessed using variance inflation factors (VIF), with VIF < 2 considered acceptable. Because MME was measured repeatedly across three positions (sitting, bipedal stance, and unipedal stance), a linear mixed-effects model (LMM) was used to account for within-subject correlation. Δ-extrusion variables were analyzed as predefined summary measures of loading-related change, whereas the linear mixed-effects model was used as a complementary repeated-measures analysis of raw MME values across positions and to test the Position × Hypermobility interaction. No formal multiplicity adjustment was applied, and secondary analyses were interpreted cautiously. Because MME was measured repeatedly in each participant across three loading conditions, a multivariable linear mixed-effects model was fitted to account for within-subject correlation. MME (mm) was entered as the dependent variable; position, hypermobility status, and their interaction (Position × Hypermobility) were specified as fixed effects; BMI, sex, age, and patellar tendon elastography were included as covariates; and participant identity was modeled as a random intercept. The Position × Hypermobility interaction was evaluated using a likelihood-ratio test. Adjusted (estimated marginal) means by position and group were derived from the fitted model. Interobserver reliability was assessed in the first 25 participants using a two-way random-effects intraclass correlation coefficient for absolute agreement [ICC (2,1)] for sitting, bipedal, and unipedal MME as well as patellar tendon elastography. A two-sided *p*-value < 0.05 was considered statistically significant for all analyses.

## 3. Results

A total of 106 healthy adults were included, exceeding the minimum required sample size of 96 participants determined by the a priori power analysis. The cohort had a balanced sex distribution and predominantly right-sided dominance, with a mean age in the late 20s and a mean BMI in the normal-to-overweight range. Generalized joint hypermobility (Beighton ≥ 5) was present in approximately one-quarter of participants (Table 2).

**Figure 4:**
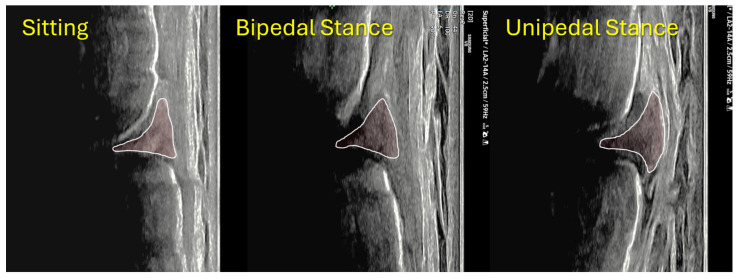
Position-dependent increase in medial meniscus extrusion (MME) across functional loading postures. Representative longitudinal (coronal-plane) ultrasound images of the medial joint line obtained in the same knee during sitting (unloaded), bipedal stance, and unipedal stance. The outlined/shaded region indicates the medial meniscal body on the standardized imaging plane. Compared with sitting, the meniscus demonstrates a progressive outward displacement (extrusion) under weight-bearing, with the greatest apparent extrusion during unipedal stance, illustrating the stepwise, load-dependent behavior of physiologic MME.

When participants were stratified by hypermobility status, the groups were comparable in age, anthropometrics, BMI, and limb dominance; however, the hypermobile group included a higher proportion of females. PT stiffness, as measured by SWE, was significantly lower in the hypermobile group. However, the hypermobile group contained a higher proportion of females, which may have influenced unadjusted between-group comparisons despite statistical adjustment for sex in the multivariable models. Across all participants, MME increased progressively from sitting (unloaded) to bipedal stance and then to unipedal stance, and this load-dependent behavior differed by hypermobility status (Figure 4). Specifically, the hypermobile group demonstrated greater MME than the normal group at each position (sitting, bipedal, and unipedal) (Table 3). In addition, the magnitude of loading-related change was larger in the hypermobile group for both Δ (bipedal − sitting) and Δ (unipedal − sitting) (Table 3).

In multivariable linear regression analyses of loading-related extrusion, PT elastography was the only independent predictor consistently associated with both Δ-unipedal and Δ-bipedal extrusion, with lower PT stiffness associated with greater increases in extrusion under load. After adjustment for age, BMI, and sex, hypermobility status was not independently associated with Δ-extrusion in either model (Table 4 and Table 5).

In the linear mixed-effects model accounting for repeated measures, position remained a strong determinant of MME, confirming a stepwise increase from sitting to bipedal and unipedal stance (Table 6). Hypermobility status was associated with higher overall MME, and there was evidence of a position-dependent effect of hypermobility: the Position × Hypermobility interaction was significant overall by likelihood-ratio testing. It was most pronounced during the unipedal stance (Table 6). Adjusted means from the mixed model showed consistently higher MME in the hypermobile group across all positions, with the best between-group separation observed under unipedal loading (Table 7).

## 4. Discussion

In this prospective ultrasound-based study of healthy adults, three principal findings were observed. First, medial meniscus extrusion (MME) increased stepwise from sitting (unloaded) to bipedal stance and further to unipedal stance, supporting the concept that meniscal position in asymptomatic knees is dynamic and load-dependent rather than fixed. Second, participants with generalized joint hypermobility showed consistently higher MME values across all tested positions and greater loading-related extrusion on unadjusted group comparisons, with the largest between-group difference observed during unipedal stance. Third, although hypermobility was associated with greater extrusion at the group level, adjusted analyses showed that lower patellar tendon stiffness on shear-wave elastography (SWE), rather than hypermobility status alone, was independently associated with greater loading-related Δ-extrusion, while the repeated-measures mixed model still demonstrated a significant Position × Hypermobility interaction. However, these findings should be interpreted with caution. In the mixed model, the interaction pattern was most clearly supported in unipedal stance, whereas the bipedal interaction term was borderline, and the elastography effect was also of borderline significance. Accordingly, the overall pattern is supportive of a load- and context-dependent relationship, but not definitively mechanistic. Taken together, these findings indicate that physiologic MME should be interpreted in relation to loading posture and individual soft-tissue mechanical characteristics rather than a single absolute threshold.

The present findings further support the view that medial meniscus extrusion (MME) is a load-sensitive, context-dependent phenomenon rather than a fixed anatomic measurement. In our cohort, MME increased in a graded manner from sitting to bipedal and then to unipedal stance, indicating a progressive loading response under functional postures. This is consistent with prior work showing that unloading may underestimate meniscal displacement and that weight-bearing assessment better reflects physiologic meniscal behavior [8,11,27]. In particular, Shimozaki et al. demonstrated increased MME under upright, full-weight loading conditions and reported strong correlations between ultrasonography and upright MRI across positions, supporting ultrasonography as a clinically practical method for functional MME assessment [11]. Likewise, Achtnich et al. emphasized that physiologic MME in healthy knees varies with loading and is influenced by host factors such as age and BMI, challenging the use of a single universal “normal” value [13]. Studies focused on weight-bearing ultrasonography have also shown acceptable reliability and reinforced the clinical feasibility of posture-dependent MME assessment in practice [28,29]. More recent work by Dey Hazra et al. further confirmed that weight-bearing increases MME in asymptomatic volunteers and highlighted the importance of testing conditions, including knee flexion angle, when interpreting extrusion values; notably, they did not observe a significant difference between one-leg and two-leg standing in their protocol [12]. In parallel, dynamic ultrasound and motion-related investigations suggest that meniscal displacement should be interpreted as a functional behavior that varies with task demands and measurement conditions, rather than as a single static value [14,16,17]. Against this background, our observation that the largest between-group separation occurred during unipedal stance—particularly in participants with generalized joint hypermobility—adds a clinically relevant nuance: higher-load testing conditions may accentuate physiologic variation rather than solely pathologic extrusion. This is especially important when interpreting commonly cited absolute thresholds (e.g., 3 mm), because physiologic loading responses in asymptomatic individuals may approach or exceed threshold-based values depending on posture, subject characteristics, and measurement protocol, as increasingly discussed in recent reviews and consensus-oriented literature [4,5,18,19,20,30].

An additional finding of the present study was that lower patellar tendon stiffness on shear-wave elastography (SWE) was independently associated with greater loading-related Δ-extrusion, whereas generalized joint hypermobility status itself did not remain an independent predictor in the adjusted regression models. This pattern may suggest that a dichotomous clinical hypermobility classification captures a broad phenotypic tendency, whereas SWE-derived tissue mechanical properties may represent one measurable correlate of the biomechanical substrate underlying load-responsive meniscal displacement. In other words, the observed hypermobility-related differences in unadjusted comparisons could be influenced, at least in part, by underlying soft-tissue compliance characteristics rather than hypermobility status per se. However, this interpretation should be made cautiously, because the regression models explained only a modest proportion of the variance in loading-related extrusion, indicating that substantial inter-individual variability remained unexplained. Although this interpretation is biologically plausible, it should be considered hypothesis-generating rather than definitive, because patellar tendon SWE is a regional measurement and not a direct assessment of meniscotibial, capsulomeniscal, or root restraint mechanics. Nonetheless, the concept is consistent with the broader literature, which indicates that meniscal extrusion is not solely a static morphologic finding but also a biomechanical behavior influenced by load transfer, restraint integrity, and functional joint conditions [4,5,15,18,30]. It is also aligned with dynamic ultrasound observations, suggesting that the magnitude and pattern of extrusion change can provide information beyond a single absolute distance measurement, particularly when interpreted across different loading states [14,16,17,27]. Accordingly, patellar tendon SWE should be interpreted as a partial and exploratory correlate of tissue mechanics rather than a dominant mechanistic determinant of extrusion behavior. From a clinical interpretation standpoint, these findings support a more cautious and individualized framework in which loading-related change and tissue mechanical context may be considered alongside hypermobility phenotype, rather than assuming that all increased extrusion in higher-load postures reflects structural meniscal pathology.

The present findings also have important implications for the interpretation of commonly used absolute MME thresholds—particularly the widely cited 3-mm cut-off—in clinical and research settings. Although threshold-based definitions are useful for standardization, accumulating evidence indicates that meniscal extrusion should not be reduced to a single static distance, as this fails to account for posture, loading conditions, knee position, and patient-specific factors [4,13,18,20,30]. In our asymptomatic cohort, mean unipedal MME in the hypermobile group exceeded 3 mm, illustrating that threshold-range values may occur under physiologic loading conditions in individuals without known meniscal pathology. This observation supports the growing view that the distinction between physiologic/paraphysiologic and pathologic extrusion should incorporate dynamic behavior rather than solely absolute magnitude. In this context, dynamic ultrasonography may offer additional interpretive value by assessing whether extrusion changes appropriately across loading states, rather than relying solely on a single measurement obtained in a single posture [14,16,17,27,28,29]. This concept is also compatible with the “dead meniscus sign” described in the setting of medial meniscus posterior root tear, in which loss of expected dynamic extrusion behavior under loading may reflect functional restraint failure despite the limitations of static threshold-based interpretation [5,9,18]. Accordingly, our results do not argue against the clinical relevance of threshold values, but rather suggest that threshold-based interpretation should be contextualized within loading protocol and dynamic response patterns, especially when evaluating asymptomatic individuals or populations with greater tissue compliance.

This study has several notable strengths. First, it was designed as a prospective observational study in a well-defined cohort of healthy adults, with standardized clinical screening to minimize confounding by occult knee pathology. Second, the ultrasound protocol was deliberately structured to capture physiologic, load-dependent behavior of the medial meniscus by comparing extrusion across unloaded sitting and progressively loaded bipedal and unipedal stance conditions, thereby addressing an essential limitation of static supine imaging. Third, measurement reproducibility was strengthened by a dedicated interobserver reliability assessment in the initial subset, demonstrating good-to-excellent agreement for extrusion across all positions and for patellar tendon SWE. Another strength of the study is the integration of dynamic meniscal extrusion assessment with quantitative patellar tendon stiffness measured by shear wave elastography, which allowed an exploratory evaluation of whether subject-specific soft-tissue mechanical characteristics may relate to loading-induced extrusion, rather than relying solely on group comparisons. The rationale for including patellar tendon SWE was not that the patellar tendon directly represents meniscotibial or capsulomeniscal restraint. Rather, it was selected as an accessible and standardized quantitative marker of subject-specific soft-tissue mechanical behavior within the same knee. In this context, lower tendon stiffness may reflect a broader pattern of increased connective tissue compliance or altered force-transmission characteristics under load, which could be associated with greater physiologic meniscal displacement. This interpretation remains indirect, however, and should be regarded as exploratory rather than mechanistically definitive. Patellar tendon SWE does not directly quantify peri-meniscal stabilizing structures, and the observed association should therefore be interpreted as a correlate of tissue mechanics rather than a direct measure of meniscal restraint. Finally, the statistical framework was aligned with the repeated-measures structure of the data: the use of Δ-extrusion metrics for clinically interpretable loading effects, multivariable regression for independent associations, and a linear mixed-effects model accounting for within-subject correlation (including a Position × Hypermobility interaction) improved internal validity and reduced bias from confounding factors such as sex, age, and BMI. In addition, focusing on asymptomatic knees enabled characterization of a physiologic reference pattern of load-responsive MME behavior, which may serve as a comparative baseline for future studies in symptomatic or degenerative populations.

Several limitations should be acknowledged. First, this was a single-center study performed in a relatively young cohort of healthy volunteers (18–40 years), which may limit the generalizability of the findings to older individuals, patients with symptomatic meniscal pathology, or populations with established osteoarthritis. Second, the definition of generalized joint hypermobility relied on the Beighton score (≥5) as a pragmatic clinical screening tool. Although widely accepted, it does not fully characterize connective tissue phenotypes. Third, medial meniscus extrusion was quantified using ultrasonography, which—despite being well suited for dynamic, weight-bearing assessment—remains operator-dependent and sensitive to technical factors such as probe orientation, probe pressure, and identification of anatomical reference points. Although we standardized transducer positioning using skin markers and confirmed good-to-excellent interobserver agreement in an initial subset, subtle variations in transducer placement between positions cannot be entirely ruled out, particularly during single-leg stance. In addition, the study design did not include MRI confirmation; therefore, asymptomatic structural meniscal variants or early degenerative changes could not be definitively ruled out, even though participants underwent a standardized clinical screening without suspicion of meniscal pathology. Fourth, the a priori sample size calculation was based on the primary between-group comparison of loading-related Δ-extrusion rather than on the Position × Hypermobility interaction term in the linear mixed-effects model; therefore, the study was not explicitly powered to test the interaction. In addition, no formal adjustment for multiplicity was applied across the multiple secondary comparisons and model terms; therefore, these findings should be interpreted with appropriate caution. Fifth, loading conditions were standardized as sitting, bipedal stance, and unipedal stance. Still, the actual magnitude of joint loading and neuromuscular control may vary between individuals, especially during unipedal stance, potentially contributing to measurement variability. In addition, residual confounding by unmeasured factors, such as physical activity level, sports participation, neuromuscular control during stance, and subtle lower-limb biomechanical differences, cannot be excluded. These factors may influence tendon stiffness, loading behavior, and dynamic meniscal mechanics. Finally, patellar tendon stiffness was used as a surrogate marker of soft-tissue mechanical behavior. At the same time, SWE provides quantitative data, and acquisition settings and residual probe compression can influence elastography values despite efforts to minimize these effects; the observed associations should therefore be interpreted as correlational rather than causal. Moreover, the modest explained variance of the regression models indicates that substantial variability remained unaccounted for, and patellar tendon SWE should therefore be interpreted as a partial correlate rather than a comprehensive mechanistic explanation of loading-related extrusion. Moreover, because the study was cross-sectional, no inference can be made regarding whether the observed “physiologic” hypermobility-related extrusion pattern is protective, neutral, or associated with future meniscal symptoms or osteoarthritic progression. Longitudinal studies are needed to clarify its prognostic significance.

## 5. Conclusions

In healthy adults, medial meniscus extrusion (MME) demonstrates a physiologic, load-dependent increase from sitting to bipedal and unipedal weight-bearing positions. Individuals with generalized joint hypermobility exhibit higher MME values across all positions and a greater load-related amplification pattern, particularly during unipedal stance. However, patellar tendon stiffness on shear-wave elastography, rather than hypermobility status itself, was independently associated with greater loading-related extrusion. This finding should be interpreted as exploratory, as patellar tendon SWE represents an indirect surrogate of tissue mechanics rather than a direct measure of peri-meniscal restraint. Overall, these results suggest that ultrasound-based MME measurements may be interpreted more appropriately when loading condition, hypermobility phenotype, and tissue mechanical context are considered alongside absolute extrusion values.

## Figures and Tables

**Table 1:** Interobserver reliability of the ultrasound measurements for the first 25 participants.

Variables	Unit	ICC (2,1)	95% CI	Interpretation
Sitting MME	mm	0.870	0.676–0.937	Good
Bipedal MME	mm	0.900	0.795–0.950	Excellent
Unipedal MME	mm	0.890	0.821–0.917	Good
PT Elastography	kPa	0.880	0.785–0.927	Good

Abbreviations: ICC: Intraclass Correlation Coefficient, CI: Confidence Interval, mm: Millimeter, kPa: Kilopascal, MME: Medial Meniscus Extrusion, PT: Patellar Tendon.

**Table 2:** Demographic characteristics of all participants.

Variables	Data
Age (years, Median, IQR)	28.0, 24.0–33.8
Sex (*n*, %)	
*Male*	51 (48.1%)
*Female*	55 (51.9%)
Dominant Side (*n*, %)	
*Right*	93 (87.7%)
*Left*	13 (12.3%)
Height (cm ± SD, range)	170.7 ± 9.4 (150.0–198.0)
Weight (kg, Median, IQR)	69.5, 59.3–79.0
BMI (kg/m^2^, Median, IQR)	23.4, 21.8–25.9
Beighton Score (score, Median, IQR)	3.0, 2.0–5.0
Hypermobility (Beighton ≥ 5) (*n*, %)	28 (26.4%)

Abbreviations: SD: Standard Deviation, BMI: Body Mass Index, IQR: Interquartile Range.

**Table 3:** Comparison of measurements between the Hypermobile and Normal groups.

Variables	Hypermobile Group (*n*:28)	Normal Group (*n*:*78*)	*p*-Value
Age, years; median (IQR, range)	29.0 (24.0–33.0, 18.0–39.0)	28.0 (24.0–34.0, 18.0–40.0)	0.744 ^1^
Sex (*n*, %)			0.004 ^2^
*Male*	7 (25.0%)	44 (56.4%)	
*Female*	21 (75.0%)	34 (43.6%)	
Dominant Side (*n*, %)			0.466 ^2^
*Right*	24 (85.7%)	69 (88.5%)	
*Left*	4 (14.3%)	9 (11.5%)	
Height (cm ± SD, range)	168.2 ± 8.7 (150.0–186.0)	171.6 ± 9.6 (152.0–198.0)	0.111 ^3^
Weight, kg; median (IQR, range)	64.5 (58.8–73.8, 47.0–120.0)	70.0 (60.0–80.0, 42.0–116.0)	0.265 ^1^
BMI, kg/m^2^; median (IQR, range)	23.5 (21.2–24.7, 18.8–39.6)	23.3 (21.9–26.3, 17.3–35.8)	0.761 ^1^
Beighton Score, score; median (IQR, range)	5.0 (5.0–6.0, 5.0–9.0)	2.0 (2.0–3.0, 0.0–4.0)	<0.001 ^1^
PT Elastography (kPa ± SD, range)	23.8 ± 7.0 (7.1–38.0)	37.6 ± 9.7 (18.9–62.8)	<0.001 ^3^
MME Sitting, mm; median (IQR, range)	1.24 (0.89–1.61, −0.18–2.75)	0.86 (0.50–1.17, −0.92–2.46)	<0.001 ^1^
MME Bipedal (mm ± SD, range)	2.36 ± 0.43 (1.38–3.23)	1.65 ± 0.58 (0.42–2.93)	<0.001 ^3^
MME Unipedal (mm ± SD, range)	3.09 ± 0.51 (2.09–4.21)	2.14 ± 0.60 (1.02–3.41)	<0.001 ^3^
Δ Bipedal−Sitting, mm; median (IQR, range)	1.06 (0.88–1.34, 0.21–2.16)	0.62 (0.40–1.27, 0.06–2.49)	0.037 ^1^
Δ Unipedal−Sitting, mm; median (IQR, range)	1.94 (1.27–2.26, 0.79–3.21)	1.19 (0.78–1.86, 0.25–3.31)	0.002 ^1^

Abbreviations: SD: Standard Deviation, BMI: Body Mass Index, IQR: Interquartile Range, MME: Medial Meniscus Extrusion, Δ (Delta): Difference. ^1^ Mann–Whitney U Test, ^2^ Chi-Square Test, and ^3^ Student *T*-Test.

**Table 4:** Multivariable linear regression analysis for Δ-unipedal MME (Unipedal − Sitting, mm).

Predictor	B (SE)	95% CI for B	*p*-Value
Age (years)	0.003 (0.012)	−0.020 to 0.027	0.772
BMI (kg/m^2^)	0.005 (0.017)	−0.029 to 0.038	0.786
Sex (male vs. female) *	−0.191 (0.146)	−0.480 to 0.098	0.193
PT elastography (kPa)	−0.024 (0.007)	−0.039 to −0.010	0.001
Hypermobility (Beighton ≥ 5)	0.076 (0.180)	−0.280 to 0.433	0.671

Predictors were entered simultaneously (enter method). * Sex coding (male = 1, female = 0). The overall regression model was significant (*p* < 0.001), explaining 20.4% of the variance in Δ-unipedal extrusion (R^2^ = 0.204; adjusted R^2^ = 0.164). Multicollinearity was not observed (all VIF < 2).

**Table 5:** Multivariable linear regression for Δ-bipedal extrusion (Bipedal − Sitting, mm).

Predictor	B (SE)	95% CI for B	*p*-Value
Age (years)	−0.002 (0.010)	−0.022 to 0.018	0.841
BMI (kg/m^2^)	−0.006 (0.014)	−0.033 to 0.022	0.692
Sex (male vs. female) *	−0.129 (0.122)	−0.370 to 0.112	0.292
PT elastography (kPa)	−0.020 (0.006)	−0.032 to −0.008	0.001
Hypermobility (Beighton ≥ 5)	−0.083 (0.150)	−0.380 to 0.214	0.580

Predictors were entered simultaneously (enter method). * Sex coding (male = 1, female = 0). The overall regression model was significant (*p* = 0.004), explaining 15.5% of the variance in Δ-bipedal extrusion (R^2^ = 0.155; adjusted R^2^ = 0.113). Multicollinearity was not observed (all VIF < 2).

**Table 6:** Linear mixed-effects model for repeated MME measurements across sitting, bipedal, and unipedal positions (*n* = 106; 318 observations). Reference: Sitting and Normal; Sex: Male = 1, Female = 0.

Fixed Effect	β	SE	95% CI	*p*-Value
Intercept	0.907	0.362	0.197 to 1.616	0.012
Position: Bipedal (vs. Sitting)	0.865	0.062	0.743 to 0.987	<0.001
Position: Unipedal (vs. Sitting)	1.358	0.062	1.236 to 1.480	<0.001
Hypermobility (Beighton ≥ 5)	0.295	0.145	0.010 to 0.579	0.042
Position (Bipedal) × Hypermobility	0.234	0.121	−0.004 to 0.471	0.054
Position (Unipedal) × Hypermobility	0.475	0.121	0.237 to 0.712	<0.001
BMI (kg/m^2^)	0.011	0.012	−0.012 to 0.035	0.340
Sex (male vs. female)	−0.111	0.103	−0.313 to 0.091	0.282
Age (years)	0.002	0.008	−0.015 to 0.018	0.834
Elastography (kPa)	−0.010	0.005	−0.020 to −0.000	0.049

**Table 7:** Interaction test and adjusted means. Likelihood-ratio (LR) test for Position × Hyper: LR = 15.076, df = 2, *p* = 0.00053. Estimated marginal means (adjusted at mean BMI/Age/Elasto and sample sex proportion).

Position	Adjusted Mean (Normal)	Adjusted Mean (Hypermobile)
Sitting	0.832	1.127
Bipedal	1.697	2.226
Unipedal	2.190	2.959

## Data Availability

The datasets are not publicly available. The de-identified data are available upon request from the corresponding author due to privacy, ethical, and legal restrictions that protect patient confidentiality.

## Abbreviations

The following abbreviations are used in this manuscript:

BMI	Body Mass Index
CI	Confidence Interval
ICC	Intraclass Correlation Coefficient
kPa	Kilopascal
LMM	Linear Mixed-Effects Model
LR	Likelihood Ratio
MCL	Medial Collateral Ligament
MME	Medial Meniscus Extrusion
MRI	Magnetic Resonance Imaging
ROI	Region of Interest
SD	Standard Deviation
SE	Standard Error
SWE	Shear-Wave Elastography
US	Ultrasonography
VIF	Variance Inflation Factor
Δ	Delta (Difference)

## References

[1] Mameri E.S., Dasari S.P., Fortier L.M., Verdejo F.G., Gursoy S., Yanke A.B., Chahla J. (2022). Review of Meniscus Anatomy and Biomechanics. Curr. Rev. Musculoskelet. Med..

[2] Kim M.S., In Y., Kim H., Jeong J., Sohn S. (2025). Why Hoop Tension Matters: A Biomechanical Perspective on Medial Meniscus Posterior Root Tears-A Narrative Review. Bioengineering.

[3] Kından Baltacı P., Toker M., Ozbek E.A. (2025). Meniscus Root Tears: Current Concepts in Anatomy, Diagnosis, and Treatment Strategies. Sports Traumatol. Arthrosc..

[4] Gajjar S.M., Solanki K.P., Shanmugasundaram S., Kambhampati S.B.S. (2021). Meniscal Extrusion: A Narrative Review. Orthop. J. Sports Med..

[5] Langhans M.T., Lamba A., Saris D.B.F., Smith P., Krych A.J. (2023). Meniscal Extrusion: Diagnosis, Etiology, and Treatment Options. Curr. Rev. Musculoskelet. Med..

[6] Swamy N., Wadhwa V., Bajaj G., Chhabra A., Pandey T. (2018). Medial meniscal extrusion: Detection, evaluation and clinical implications. Eur. J. Radiol..

[7] Tortorella F., Boffa A., Di Martino A., Andriolo L., Facchini G., Di Carlo M., Miceli M., Zaffagnini S., Filardo G. (2024). Meniscal Extrusion Correlates with Symptom Severity in Knee Osteoarthritis: An Ultrasound and Magnetic Resonance Imaging Analysis of 100 Patients. J. Clin. Med..

[8] Nogueira-Barbosa M.H., Gregio-Junior E., Lorenzato M.M., Guermazi A., Roemer F.W., Chagas-Neto F.A., Crema M.D. (2015). Ultrasound assessment of medial meniscal extrusion: A validation study using MRI as reference standard. AJR Am. J. Roentgenol..

[9] Karpinski K., Diermeier T., Willinger L., Imhoff A.B., Achtnich A., Petersen W. (2019). No dynamic extrusion of the medial meniscus in ultrasound examination in patients with confirmed root tear lesion. Knee Surg. Sports Traumatol. Arthrosc..

[10] Johnson S.E., Kruse R.C., Boettcher B.J. (2024). The Role of Ultrasound in the Diagnosis and Treatment of Meniscal Injuries. Curr. Rev. Musculoskelet. Med..

[11] Shimozaki K., Nakase J., Oshima T., Asai K., Toyooka K., Ohno N., Miyati T., Tsuchiya H. (2020). Investigation of extrusion of the medial meniscus under full weight-loading conditions using upright weight-loading magnetic resonance imaging and ultrasonography. J. Orthop. Sci..

[12] Dey Hazra M.E., Dey Hazra R.O., Kruse A., Bradshaw A., Hollenbeck J., Millett P.J., Vidal A., Tashman S., Watkins L.E. (2025). Physiologic Medial Meniscal Extrusion with Loading and Flexion Detected by Ultrasound. Orthop. J. Sports Med..

[13] Achtnich A., Petersen W., Willinger L., Sauter A., Rasper M., Wörtler K., Imhoff A.B., Diermeier T. (2018). Medial meniscus extrusion increases with age and BMI and is depending on different loading conditions. Knee Surg. Sports Traumatol. Arthrosc..

[14] Hashizume T., Ishii Y., Nakashima Y., Okamoto S., Iwamoto Y., Okada K., Takagi K., Adachi N., Takahashi M. (2023). Evaluation of meniscus extrusion during stair ambulation in healthy volunteers using dynamic ultrasonography: A feasibility study. J. Med. Ultrason..

[15] Crema M.D., Roemer F.W., Felson D.T., Englund M., Wang K., Jarraya M., Nevitt M.C., Marra M.D., Torner J.C., Lewis C.E. (2012). Factors associated with meniscal extrusion in knees with or at risk for osteoarthritis: The Multicenter Osteoarthritis study. Radiology.

[16] Bendak S.F., Georgii J., Taghizadeh E., Heldmann S., Meine H., Lange T., Buchholtz J., Fuchs A., Mayr M., Schmal H. (2025). In vivo dynamic intrusion and extrusion of the menisci in varus and valgus load within a healthy population. J. Exp. Orthop..

[17] Meng X.Y., Li Z.Q., Ding H.F., Wang D.Y., Dai L.H., Jiang D. (2024). A Novel Ultrasound Method of Evaluating Dynamic Extrusion of Lateral Meniscus in Healthy Population: Different Patterns of Dynamic Extrusion Revealed Between Lateral and Medial Meniscus. J. Ultrasound Med..

[18] Martinese G., Tortorella F., Andriolo L., Facchini G., Miceli M., Filardo G. (2025). Ultrasound quantification of knee meniscal extrusion: The potential of weight-bearing and dynamic evaluations. A systematic review. EFORT Open Rev..

[19] Boksh K., Shepherd D.E.T., Espino D.M., Shepherd J., Ghosh A., Aujla R., Boutefnouchet T. (2024). Assessment of meniscal extrusion with ultrasonography: A systematic review and meta-analysis. Knee Surg. Relat. Res..

[20] Barreira F., Gomes E., Oliveira S., Valente C., Bastos R., Sánchez M., Andrade R., Espregueira-Mendes J. (2023). Meniscal extrusion in knees with and without osteoarticular pathology: A systematic review of normative values and cut-offs for diagnostic criteria. Knee.

[21] Birinci M.C., Makaracı Y., Aktaş B.S., Atasever G., Ruiz-Cárdenas J.D. (2026). The single-leg sit-to-stand test is valid and reliable for assessing lower limb performance and asymmetry in international cross-country skiers. Gait Posture.

[22] Malfait F., Francomano C., Byers P., Belmont J., Berglund B., Black J., Bloom L., Bowen J.M., Brady A.F., Burrows N.P. (2017). The 2017 international classification of the Ehlers-Danlos syndromes. Am. J. Med. Genet. Part C Semin. Med. Genet..

[23] Reuter P.R., Fichthorn K.R. (2019). Prevalence of generalized joint hypermobility, musculoskeletal injuries, and chronic musculoskeletal pain among American university students. PeerJ.

[24] Kawaguchi K., Enokida M., Otsuki R., Teshima R. (2012). Ultrasonographic evaluation of medial radial displacement of the medial meniscus in knee osteoarthritis. Arthritis Rheumatol..

[25] Kot B.C., Zhang Z.J., Lee A.W., Leung V.Y., Fu S.N. (2012). Elastic modulus of muscle and tendon with shear wave ultrasound elastography: Variations with different technical settings. PLoS ONE.

[26] Berrigan W.A., Cipriano K., Easley K.A., Mautner K. (2025). Quantifying Mechanical Properties of the Patellar and Achilles Tendons Using Ultrasound Shear Wave Elastography: A Pilot Study. Diagnostics.

[27] Reisner J.H., Franco J.M., Hollman J.H., Johnson A.C., Sellon J.L., Finnoff J.T. (2021). The Difference in Medial Meniscal Extrusion between Non-Weight-Bearing and Weight-Bearing Positions in People with and without Medial Compartment Knee Osteoarthritis. PM R.

[28] Reisner J.H., Franco J.M., Hollman J.H., Johnson A.C., Sellon J.L., Finnoff J.T. (2020). Ultrasound Assessment of Weight-Bearing and Non-Weight-Bearing Meniscal Extrusion: A Reliability Study. PM R.

[29] Falkowski A.L., Jacobson J.A., Cresswell M., Bedi A., Kalia V., Zhang B. (2022). Medial Meniscal Extrusion Evaluation with Weight-Bearing Ultrasound: Correlation with MR Imaging Findings and Reported Symptoms: Correlation with MR Imaging Findings and Reported Symptoms. J. Ultrasound Med..

[30] Familiari F., Chahla J., Compagnoni R., DePhillipo N.N., Moatshe G., LaPrade R.F., Meniscus International Network (MenIN) Study Group (2024). Meniscal extrusion consensus statement: A collaborative survey within the Meniscus International Network (MenIN) Study Group. Knee Surg. Sports Traumatol. Arthrosc..

